# DYNet: A Printed Book Detection Model Using Dual Kernel Neural Networks

**DOI:** 10.3390/s23249880

**Published:** 2023-12-17

**Authors:** Lubin Wang, Xiaolan Xie, Peng Huang, Qiang Yu

**Affiliations:** 1 Institute of Information Science and Engineering, Guilin University of Technology, Guilin 541004, China; 2120211049@glut.edu.cn (L.W.); xxl@glut.edu.cn (X.X.); 2016014@glut.edu.cn (P.H.); 2 National Space Science Center, Chinese Academy of Sciences, Beijing 100190, China

**Keywords:** small-target detection, high-precision detection, convolutional neural networks

## Abstract

Target detection has always been a hotspot in image processing/computer vision research, and small-target detection is a frequently encountered problem in the field of target detection. With the continuous innovation of target detection technology, people always hope that the detection of small targets can reach the real-time accuracy of large-target detection. In this paper, a small-target detection model based on dual-core convolutional neural networks (CNN) is proposed, which is mainly used for the intelligent detection of books in the production line of printed books. The model is mainly composed of two modules, including a region prediction module and suspicious target search module. The region prediction module uses a CNN to predict suspicious region blocks in a large context. The suspicious target search module uses a different CNN from the above to find tiny targets in the predicted region blocks. Comparative testing of four small book target samples using this model shows that this model has better book small-target detection accuracy compared to other models.

## 1. Introduction

Books are crucial to human society as an important medium for cultural inheritance and knowledge accumulation. However, due to the differences in printing equipment and processes of different printing manufacturers, the quality of book printing is often highly variable, affecting the quality of books and reading experience of readers. Therefore, intelligent book printing quality detection is an important research direction. This study was aimed at the intelligent detection and evaluation of book quality through technical means, such as computer vision and image processing.

The post-printing processes of book production include folding, assembling, and binding. The quality control of each process is particularly important. In the detection of book ladder labels, the traditional single template matching algorithm cannot meet the requirement for accurate detection because the book ladder label has a small area in the whole picture and the size is inconsistent [1]. In 1992, Gengfeng et al. [2] proposed the gray-code color code detection method based on a single-board microcomputer. In 2009, Fei [3] developed a book sticker detection system based on machine vision. In 2014, Xiaoqing [4] developed a total and collator detection system for the children’s hardcover book production line. The above algorithms and systems all used a single matching algorithm. This type of algorithm can detect whether more or less book stickers were attached, but there were still serious issues, such as missed or false detections.

The above issues can be addressed to some extent through edge detection and image registration techniques. In 2016, Mei and Xiangmin [5] developed an online inspection system for ladder labels during book binding. The system binarized the image and used the Roberts operator for edge detection, followed by similarity matching for registration. In 2018, Guo and Xinwen [6] proposed an adaptive ladder label detection method based on phase correlation. By using the phase correlation registration technology, the phase correlation between the ladder label video frame and template ladder label was calculated, and two frames of images were registered to determine whether there were errors in the assembly. These two algorithms required high image quality with low image noise. When the image resolution was low, there were still serious problems, such as missed or false detection. In practical production, serious quality problems often occur, causing certain economic losses. Thus, they cannot be used in production practice. In 2021, Lihong [7] designed a book assembly matching method. This method can be used to perform book label repair, book label recognition, and shape matching cost calculation based on the results of book label detection and lock line region segmentation. However, for the lock line region segmentation method, if there were too serious phenomena, such as burrs, protrusions, or dents, the number of iterations for edge smoothing would increase, thereby increasing the processing time. Therefore, it cannot meet the requirements of real-time detection in the production line.

**Figure 1:**
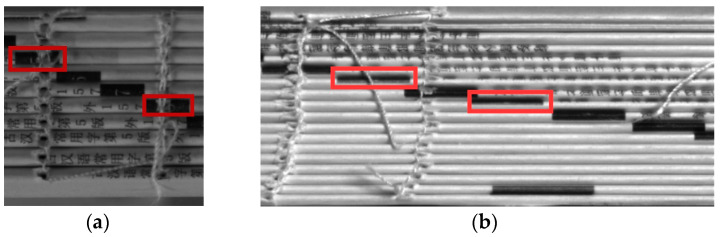
Examples of ladder labels that are difficult to detect. (**a**) The ladder label marked by the red box was bisected by the binding line. (**b**) The size of the ladder label marked by the red box was one third of the normal ladder label.

In recent years, there have been many breakthroughs in target detection algorithms based on the convolutional neural network (CNN) in the field of deep learning. These CNN-based target detection algorithms have achieved superior results in target detection. Compared with traditional target detection algorithms, CNN-based target detection algorithms can integrate different tasks, such as feature extraction, feature fusion, and feature classification, into the same network [8]. Using CNNs for book ladder label detection has become more common in the field of book detection. In 2022, Huabin et al. [9] designed a book ladder label detection algorithm. The method was improved based on the YOLOv5 detection network. By modifying the network structure, the detection accuracy of book ladder labels by the network was improved. In 2023, Shi and Qiang [10] designed a YOLO-based automated bookbinding detection system. The system used YOLOv3 for recognition and then used numerical fitting to determine the number of identified label blocks. The combined judgment based on these two approaches was used as the basis for determining whether the book sample was bound incorrectly. However, in the actual production process, the binding process often results in the ladder labels in the samples being split in half by the binding line, as shown in Figure 1a. Recognizing such samples as two targets in this detector led to low recall rates and frequent false alarms. In some cases, the width of the ladder label may become one-third that of the normal ladder label, as shown in Figure 1b. The algorithm had low detection accuracy for these ladder labels and often led to missed detections. Although the detection accuracy of this algorithm reached the level required for actual production, the low recall rates often led to quality issues in sold products. Therefore, it is urgent to improve the recall rate of the book ladder label detection algorithm.

In contrast, in recent years, in order to achieve the accurate and rapid detection of objects in images, many scholars have proposed a variety of deep learning methods, which play a key role in the field of target detection, and an in-depth study of this will help to better characterize the method proposed in this paper.

First, the SSD (Single Shot MultiBox Detector [11]) is a popular target detection method that achieves high detection performance by using feature maps at different scales and using default boxes for each feature map location for regression and classification.

Second, the YOLO (You Only Look Once) family is another important group of target detection algorithms. The original YOLOv3 [12] introduced three scales of detection and used multi-scale prediction and three different sizes of anchors to improve the model’s detection of small objects. YOLOv4 [13] further improved the detection performance by proposing the use of clustered anchor points, an improved feature extractor, and a PANet that uses an attention mechanism, which make the performance and efficiency stand out among many target detection models.

YOLOv5s, a new member of the YOLO family, optimizes the size and speed of the model so that it can run on devices with more limited resources while maintaining higher accuracy.

YOLOv7 [14] is a lightweight real-time target detection algorithm proposed by the authors of YOLOv4. YOLOv7 is not only able to achieve better performance on large-scale, multi-class datasets, but is also able to train and detect effectively on small-sample, small-class datasets.

In conclusion, the traditional template matching-based method has problems such as missed and false detections in ladder label detection. Although the edge detection and image registration techniques present improvements in terms of detection accuracy, they have high requirements for image quality. The traditional YOLO-based detection network has improved detection accuracy, but it still has difficulty identifying certain ladder labels.

To address the difficulties of book ladder label detection, this study designed a high-precision detection model called double YOLO net (DYNet) for small targets. It combined the extraction of object regions with the classification and recognition of regions into one model. A comparative experiment revealed that compared with other detection models, DYNet had higher detection accuracy and could realize the real-time, fast, and accurate detection of book ladder labels. Thus, it could be applied to improve the efficiency of the actual book production line.

## 2. Principles and Methods

This study considered candidate region-based object detection algorithms and found that there were two stages: candidate region generation and object classification. In the candidate region generation stage, the candidate regions cover different parts of the input image using rectangular boxes of different sizes and shapes [15]. In the target classification stage, the algorithm classifies these candidate regions and gives the location and category of the target. Generally, selective search algorithms [16] or some improved algorithms (such as SQR [16], RetinaRPN [17], and MCG [18]) are used to generate candidate regions that may contain targets. However, selective search algorithms require a significant amount of time and computational resources and generate a large number of candidate regions, and most candidate regions are redundant, which can affect the accuracy of target detection.

Therefore, in this paper, a CNN-based regional prediction module (RPM) is proposed for candidate region generation. Due to the intervention of artificial supervised learning and the characteristics of CNNs, only one calculation is needed to ensure that there are always targets in the region, which can greatly reduce the occurrence of “false positives”.

Afterwards, the predicted area output from RPM is input into the suspicious target search module (STSM). The image is processed through the noise reduction algorithm module (NRAM) before input. For the original image, the target to be detected is small. However, for the output predicted area after RPM processing, it is a large or medium-sized target. By using a detection framework that performs well in detecting large objects, it is easier to obtain the desired results. As a result, certain target detection frameworks are modified to make them more suitable for detecting the desired targets.

**Figure 2:**
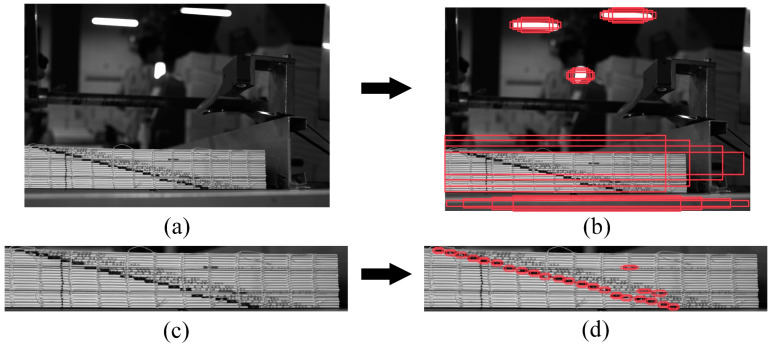
(**a**) Image taken by the camera. (**b**) Output image after RPM prediction: the red box in the figure is the suspicious target area predicted by RPM. (**c**) Output image obtained by NRAM cropping after RPM output, which is used as the input image of STSM. (**d**) Output image of STSM: the red box in the figure shows the final predicted book ladder markers.

The DYNet proposed in this paper mainly consists of two parts: RPM and STSM. Between the RPM and STSM, the NRAM is added to reduce image noise, and a quality evaluator (QE) is designed to evaluate the detection performance of this network. The input and output results of each module are shown in Figure 2.

In terms of dataset acquisition, this study uses a Dahua industrial camera with a photoelectric sensor to take pictures of books on the production line of some printing factories, and the camera automatically takes pictures when the books pass the photoelectric sensor, and a total of about 11,000 images are acquired. After eliminating some blurred images or images with shifted shooting angles due to camera vibration, we obtain about 10,000 usable images, 7000 images for the training set, and about 2000 images for the validation set. The final effect is tested on a factory assembly line with a total of 3342 books. The source of the test set and the test results will be presented in Section 5, the Experimental Results section.

## 3. DYNet Model

### 3.1. RPM

**Figure 3:**
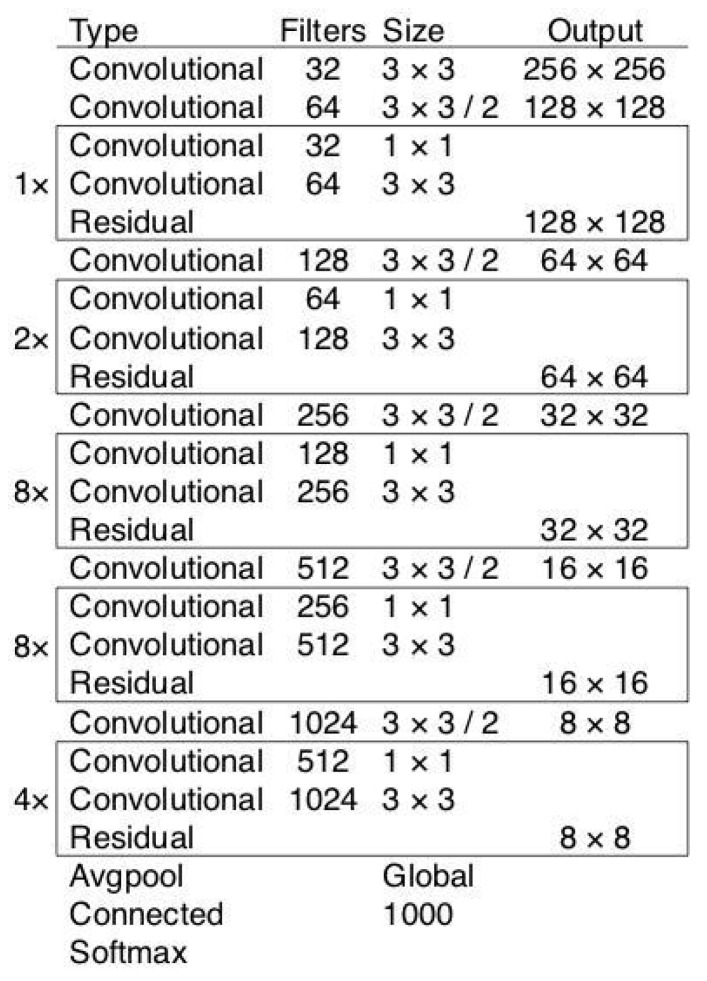
CSPDarknet-53 convolutional neural network structure.

Due to the high requirements for detection accuracy and speed, a target detection network with CSPDarknet-53 [19] as the core was chosen. As shown in Figure 3, CSPDarknet-53 is based on Darknet-53 and adds a CSP convolution structure to the original neural network, which improves the detection accuracy and speed. It is a target detection network with high accuracy and speed.

This study simplified part of the backbone of the network based on industrial production requirements. Due to the large search area, the dataset used can be considered as a large-scale object detection dataset. Also, the search area was roughly the same; thus, even reducing the number of convolutional layers to less than 20 could achieve the expected effect.

#### 3.1.1. Network Structure

**Figure 4:**
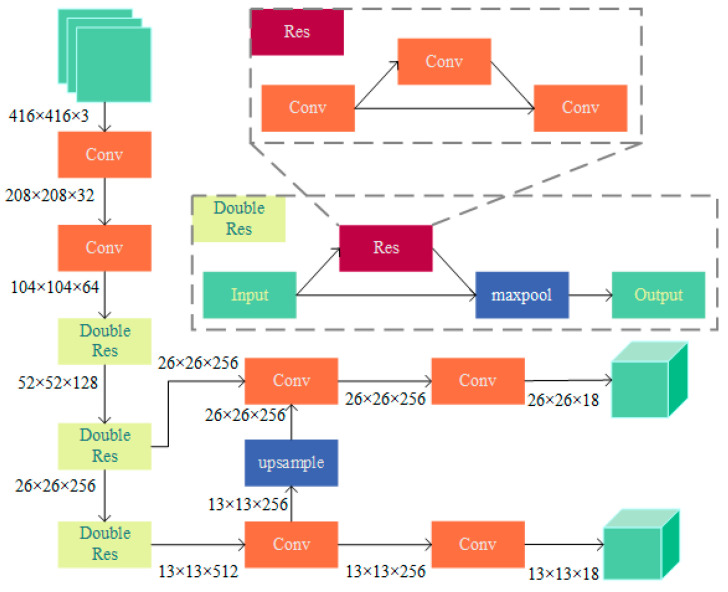
RPM network structure.

RPM is a lite version of CSPDocknet-53, with an overall structure of 19 convolutional layers, which greatly improve the detection speed. Figure 4 shows the detailed structure of the RPM network. The prediction branch was removed from the original network, and the single residual structure was changed to a double residual structure.

The final output of RPM consists of a quintet *loss_RPM_, x, y, w, h*, where the quartet $\left(x,y,w,h\right)$ is the center coordinates of the detected object (x,y) as well as the height and width of the object $\left(w,h\right)$; and the ${loss}_{RPM}$ is the loss of the final output of the RPM, which consists of two parts as shown in the following equations, where $\lambda_{1}$ and $\lambda_{2}$ are the balancing coefficients.

$${loss}_{RPM}=\lambda_{1}\mathrm{Pr}\left(obj\right)+\lambda_{2}\mathrm{Pr}\left(loc\right)$$

One of them is the confidence loss of the object, Pr(obj), which indicates the probability that the detected object is a real object, as shown in the following equation, where $p_{c}$ is the probability that the detected object is a real object.

$$\mathrm{Pr}\left(obj\right)=-ln\left(p_{c}\right)$$

The second is the coordinate loss $\mathrm{Pr}\left(loc\right)$, which represents the difference between the coordinates of the detected object and the center of the real object, as shown in the following equation, where b denotes the real coordinates and $\hat{b}$ denotes the predicted coordinates.

$$\mathrm{Pr}\left(loc\right)={[\left(b_{x}-{\hat{b}}_{x}\right)}^{2}+{\left(b_{y}-{\hat{b}}_{y}\right)}^{2}+{\left(b_{w}-{\hat{b}}_{w}\right)}^{2}+{\left(b_{h}-{\hat{b}}_{h}\right)}^{2}]$$

#### 3.1.2. Double Residual Structure

CNNs can learn more fine-grained features that help to localize the target in shallow convolutional structures and more semantic features that help to classify the target in deep convolutional structures [20]. Therefore, it was possible to use the reduction of residual structures and stacking of residual layers in the RPM to improve the detection speed.

**Figure 5:**
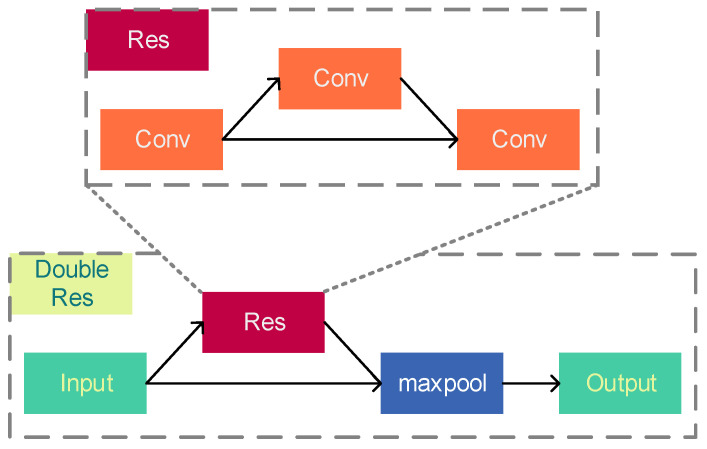
Double residual structure.

Three double residual structures were used in the RPM, as shown in Figure 5. This structure allows output features at different depths in the network to learn from each other and perform feature fusion between features with different depth resolutions. This not only improves detection accuracy compared to simply stacking convolutional kernels but also solves the problem of slow detection speed caused by multiple residual structures [21].

### 3.2. STSM

Unlike RPM, the main task of STSM is to detect small targets, so the network structure should be deepened, and some new features should be added to obtain better detection performance.

#### 3.2.1. Network Structure

**Figure 6:**
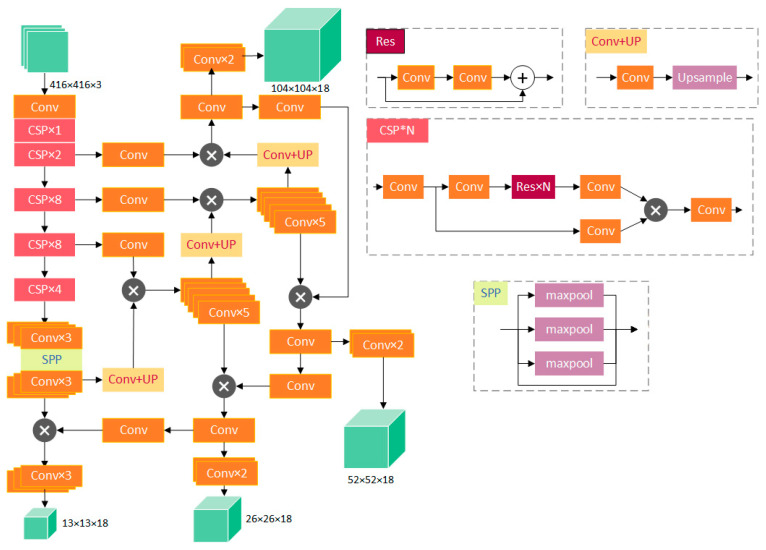
STSM network structure.

To achieve better detection performance, the network structure of CSPParknet-53 was modified to be more suitable for detecting ladder labels at the spine of printed books, as shown in Figure 6. On the basis of the original network, a prediction branch was added, and the Mish activation function was chosen. The modified network was named STSM.

The final output of STSM is consistent with RPM, which consists of a quintuple $\left({loss}_{STSM},x,y,w,h\right)$, but the loss function of STSM, ${loss}_{STSM}$, consists of three parts. When the size of the input image is S×S×B, the output of ${loss}_{STSM}$ is detected by STSM as shown in the following equation, and the STSM will iterate over all. The prediction frame will sum up the three parts of the loss with specific weights, where $\lambda_{3}$, $\lambda_{4}$, and $\lambda_{5}$ are the balancing coefficients, which will be used to obtain the most suitable value of the STSM when it is trained.

$${loss}_{STSM}=\sum_{i=0}^{S^{2}}\sum_{j=0}^{B}{[\lambda }_{3}{\mathrm{Pr}}_{\mathrm{ij}}\left(obj\right)+\lambda_{4}{\mathrm{Pr}}_{\mathrm{ij}}\left(loc\right)+\lambda_{5}{Pr}_{ij}(IoU)]$$

The two parts in ${loss}_{STSM}$ are the same as RPM, which are the confidence loss Pr(obj) and coordinate loss $\mathrm{Pr}\left(loc\right)$ of the object.

**Figure 7:**
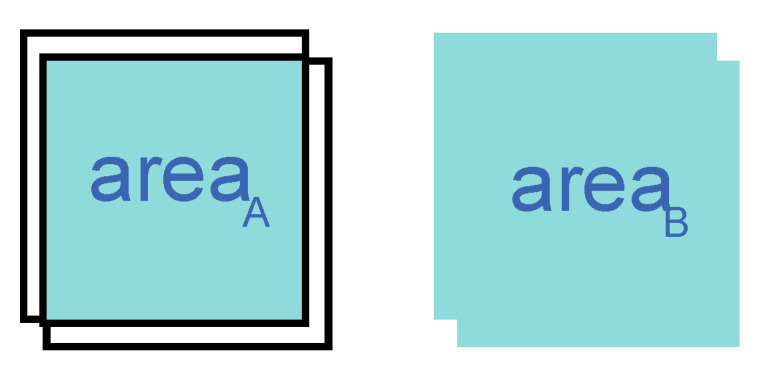
IoU definition: the ratio of intersection area to union area.

The other part is IoU [22] loss $\mathrm{Pr}\left(IoU\right)$; IoU is Intersection over Union (IoU), as shown in the following equation and Figure 7; ${area}_{A}$ is the Intersection over Union of two targets recognized by STSM; ${area}_{B}$ is the Intersection over Union of two targets recognized by STSM.

$$\mathrm{Pr}\left(IoU\right)=1-\frac{\left|{area}_{A}\right|}{\left|{area}_{B}\right|}$$

#### 3.2.2. Mish Activation Function

The difference compared to RPM replaces the LeakyReLU function used by RPM and uses Mish [23] as the activation function, with the function expression shown in the following equation.

$$f\left(x\right)=xtanh\left(\ln\left(1+e^{x}\right)\right)$$

where x is the input, and $f\left(x\right)$ is the output. In the experiment, it was found that compared to the LeakyReLU function, the Mish function had different smoothing properties, which gave it better generalization capability. This generalization capability can enable the function to effectively optimize the results of ladder label detection and improve the accuracy of detection.

#### 3.2.3. Multi-Target Detection Branch

In the dataset used in this study, the width of a few ladder labels may in some cases become one-third the width of normal ladder labels. In the target detection framework, when outputting feature maps, the following equation can be used:

$$f_{i}^{l}=\left\{\begin{array}{c} \sigma \left(W_{i}^{l}f_{i}^{l-1}+b_{i}^{l}\right),\;\;l\leq L-1\\ W_{i}^{l}f_{i}^{l-1}+b_{i}^{l},\;\;l=L \end{array}\right.$$

where $f_{i}^{l}$ denotes the feature map of the ith detection branch in the lth layer; $W_{i}^{l}$ and $b_{i}^{l}$ denote the weight and bias of the ith detection branch in the lth layer, respectively; σ denotes the activation function; and L denotes the total number of layers of the network. The more detection branches the final output has, the more feature information can be extracted in the deep layers of the network, thereby improving the detection performance for small targets but also increasing the time complexity of network computation.

The experimental comparison showed that using three detection branches led to a lack of detection accuracy. However, when there were too many detection branches, “false positives” may occur. Therefore, to detect these smaller ladder labels and balance the detection time and accuracy, one detection branch was added after the original three detection branches. Thus, STSM had four detection branches and was equipped with better detection performance for these small targets.

### 3.3. NRAM

Due to the low brightness and high noise of certain samples in the self-built dataset, the edges of the target to be detected were difficult to distinguish from the background. Therefore, it was necessary to use an image filtering algorithm to visibly separate the edges of the ladder markers, making it easier for the detection algorithm to recognize the ladder markers. When dealing with image noise, the edge feature information of the object should be preserved, but the pixel values near the edges needed to be processed. Bilateral filtering is a Gaussian filtering function based on spatial distribution. Near the edges, the pixels that are farther away have less effects on the pixel values of the edges. Thus, the edges were more prominent after processing the image using the bilateral filtering algorithm. The mathematical expression is as follows:

$$g\left(x,y\right)=\frac{1}{W_{p}}\sum_{y\in \omega }I\left(y\right)f\left({\left\|I\left(x\right)-I\left(y\right)\right\|}^{2}\right)g\left({\left\|x-y\right\|}^{2}\right)$$

where $g\left(x,y\right)$ denotes the pixel value of the filtered image at location x; $I\left(y\right)$ denotes the pixel value of the original image at location y; ω is a fixed-size neighborhood centered at x; $W_{p}$ is the normalization coefficient, i.e., the value of the filter summation; and f and g are weight functions based on the telemetry and neighborhood distances, respectively. The weight function f was used to calculate the gray scale difference between individual pixels in the neighborhood, while g was used to calculate the distance between the neighboring and current pixel points.

Although bilateral filtering can better handle pixels at the edges, it cannot perform overall noise reduction of the image effectively. Therefore, an image filtering module of bilateral filtering combined with mean filtering was used in the process of bridging RPM and STSM to reduce image noise and preserve edge features. The mathematical expression is as follows:

$$g\left(x,\;y\right)=\frac{1}{K^{2}}\sum_{u=x-\frac{K-1}{2}}^{x+\frac{K-1}{2}}\sum_{v=y-\frac{K-1}{2}}^{y+\frac{K-1}{2}}f\left(u,\;v\right)$$

where $g\left(x,y\right)$ denotes the filtered image, $f\left(u,v\right)$ denotes the input image, and K is the filter size.

During the experiments, the noise reduction module was outstandingly effective in reducing the noise of the dataset samples.

## 4. Quality Evaluator

**Figure 8:**
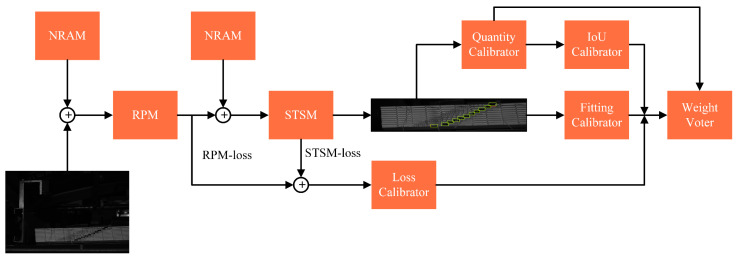
The role of quality evaluator in DYNet.

In this study, among various types of detectors, it was found that, due to the existence of some ladder labels that were difficult to detect, it was difficult to accurately assess the performance of detectors on special samples using a single evaluation index. Thus, to verify the robustness of the proposed model, a quality evaluator was designed to evaluate the recognition level of various detectors on the experimental samples. In QE, there was no significant error in the detection accuracy of various detectors for general samples, but there was a significant difference in the detection accuracy for special samples. The process of QE in DYNet is shown in Figure 8. QE consists of the following modules: quantity checker, integrated checker, and weight voter. The integrated checker consists of three modules: fitting checker, IoU checker, and loss checker.

### 4.1. Quantity Checker

The quantity checker compared the quantity of targets detected by STSM with the prestored quantity to determine the degree to which the targets were correctly recognized. This module was mainly used for targets with larger intervals after being cut by the binding line, and the calculation process is as follows:

$$QC=\left\{\begin{array}{c} 0,\;num\neq conf\\ 1,\;num=conf \end{array}\right.$$

where QC is the output of the quantity checker, num is the number of targets detected by STSM, conf is the number in the configuration file, and QC is 1 when num=conf and 0 when num≠conf.

### 4.2. Fitting Checker

The fitting checker was used to compare the target coordinates with prestored parameters after linear fitting. Due to the linear characteristics of the target itself, the module could easily evaluate the translocated target, and the evaluation indicator used for the fitting checker was the correlation coefficient, defined as follows:

$$FC=r\left(x,y\right)=\frac{Cov\left(x,y\right)}{\sqrt{Var\left[x\right]Var\left[y\right]}}$$

where FC is the output result of the fitting checker, $Cov\left(x,\;y\right)$ is the covariance between x and y, $Var\left[x\right]$ is the variance of x, and $Var\left[y\right]$ is the variance of y. The larger $\left|FC\right|$ is, the better the effect of the fitting checker.

### 4.3. IoU Checker

The IoU calibrator used the CIoU [24] calculation and took a threshold upper limit of 0.35 to evaluate targets that were less spaced after being cut by the binding line, with the defining equation shown in the following equation.

$$IC=\mathrm{Pr}\left(IoU\right)-(\frac{\rho^{2}\left(b_{x},b_{y},\;{\hat{b}}_{x},\;{\hat{b}}_{y}\right)}{c^{2}}+\alpha v)$$

where IC is the IoU calibrator output, which is calculated using the IoU loss $\mathrm{Pr}\left(IoU\right)$ output from the STSM; c is the minimum diagonal distance of the closure region that can contain both the predicted target and the real target; $\rho \left(b_{x},b_{y},\;{\hat{b}}_{x},\;{\hat{b}}_{y}\right)$ is the Euclidean distance between the centroid of the predicted target and the real target; α is the weighting parameter; and v is used to measure the similarity of aspect ratio. The specific definition is shown in the following equation, where $b_{x}$, $b_{y}$, $b_{w}$, $b_{h}$ are the coordinates of the real target, and ${\hat{b}}_{x}$, ${\hat{b}}_{y}$, ${\hat{b}}_{w}$, ${\hat{b}}_{h}$ are the coordinates of the predicted target output by STSM.

$$\left\{\begin{array}{c} \rho \left(b_{x},b_{y},\;{\hat{b}}_{x},\;{\hat{b}}_{y}\right)=\sqrt{{\left(b_{x}-{\hat{b}}_{x}\right)}^{2}-{\left(b_{y}-{\hat{b}}_{y}\right)}^{2}}\\ \alpha =\frac{v}{[1-\mathrm{Pr}\left(IoU\right)]+v}\\ v=\frac{4}{\pi^{2}}{\left[\arctan\left(\frac{b_{w}}{b_{h}}\right)-\arctan\left(\frac{{\hat{b}}_{w}}{{\hat{b}}_{h}}\right)\right]}^{2} \end{array}\right.$$

### 4.4. Loss Checker

The change that occurred in the loss checker is a weighted average of the RPM and STSM outputs, which can be expressed as follows:

$$LC=\frac{{5\times Loss}_{RPM}+2\times {Loss}_{STSM}}{7}$$

where LC is the loss checker output, ${Loss}_{RPM}$ is the value of the LeakyReLU loss function used in RPM, and ${Loss}_{STSM}$ is the value of the Mish loss function used in STSM.

### 4.5. Weighted Voting Rights

The weighted voting rights accumulated the above modules with different weights and voted. The voting results were included in the final evaluation indicators. In the weighted voter, the final experimental results can be expressed as follows:

$$WV=\frac{QC}{1+e^{-\left(0.6\times FC+IC+0.5\times LC\right)}}$$

where WV is the final output result. A WV close to 1 indicates that DYNet performed better in detecting samples, while a value close to 0 indicates that the quantity of ladder labels did not match the actual quantity.

## 5. Experimental Results

### 5.1. Datasets and Assessment Indicators

The four small-target sample comparison tests in this study used a total of 3342 images with a range of 10 to 30 small targets. These small-target sample datasets were collected on the production line of a printing factory, including the ladder labels for 11 types of books. The scene covered all the complex on-site backgrounds at the printing plant, including lighting intensity and on-site noise level, as well as some human random errors, such as deliberately rotating the book at random angles, which were crucial for our study. The dataset was obtained from reliable and representative sources and was properly cleaned and processed to ensure its quality and accuracy.

**Figure 9:**
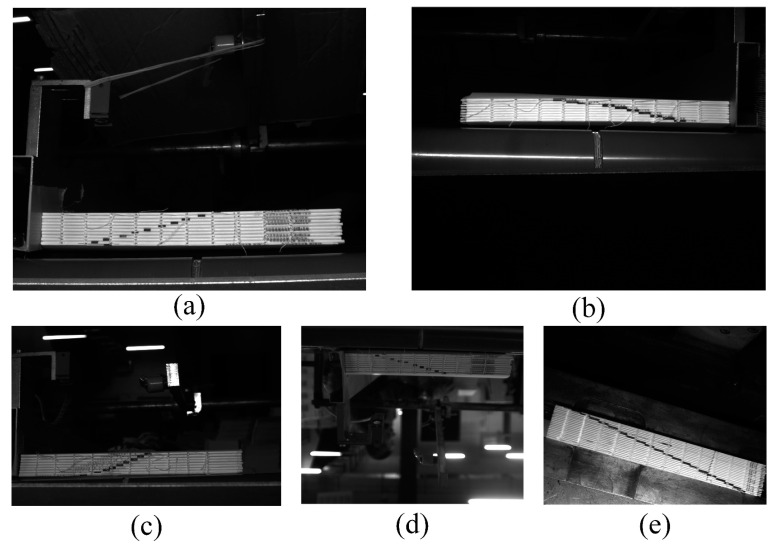
Examples of small-target samples: (**a**) Sample 1. (**b**) Sample 2. (**c**) Sample 3. (**d**) Sample 4. (**e**) Sample placed manually and randomly.

Examples of the four samples are shown in Figure 9, with the red boxes showing the locations of the ladder labels, Table 1 describing the details of these small-target samples, and Table 2 describing the source of these small-target samples.

It is common to use the average precision (AP) and average recall (AR) to evaluate how well a model matches the dataset:

$$AP=\frac{TP}{TP+FP}$$

$$AR=\frac{TP}{TP+FN}$$

where TP is the correctly identified target, FP is the target that is not recognized, and FN is the object that is not a target but is recognized as a target. The accuracy reflects how often the target identified by the model is the real target. The higher the accuracy, the better the detection performance of DYNet for samples. The recall rate is the level at which the real target is recognized by the model. It is based on the situation where the IoU value between the predicted box and real box is greater than 0.35 to determine that the predicted object was the real target. The higher the recall rate, the better DYNet can identify the real sample and the fewer samples were missed.

In this study, we used the output of QE as the basis for evaluating the performance of various types of detectors. When the result of the QE output, i.e., WV, was approaching 1, it indicated that the target recognized by DYNet was more accurate. In this study, we took the threshold value WV=0.8 as the evaluation indicator. When WV≥0.8, we considered the target as TP and when WV<0.8, we considered the target as FN.

### 5.2. Experimental Process

The experiments were conducted on a 3070 Ti GPU (Colorful iGame GeForce RTX 3070 Ti Vulcan OC, From Shenzhen, Guangdong, China), and the experimental process used the Darknet framework to train the model with a training set of 7000 images.

Through tuning optimization, the base learning rate was set to 0.0015 in this study, the input resolution was set to 416 × 416, and in order to match the resolution, compression and stretching operations were performed on the images.

Some data augmentation operations were used to generate more training samples. In this case, the rotation angle was set to 1.5, the saturation adjustment parameter was set to 1.5, the exposure adjustment parameter was set to 1.5, and the tone adjustment parameter was set to 1.5.

### 5.3. Ablation Experiments

To validate the impact of each module in DYNet, ablation experiments were performed on four small-target samples, as shown in Table 3. Comparative experiments were conducted on networks with and without RPM and then on networks with and without NRAM. As STSM is a necessary detection module, it was used in all ablation experiments.

**Figure 10:**
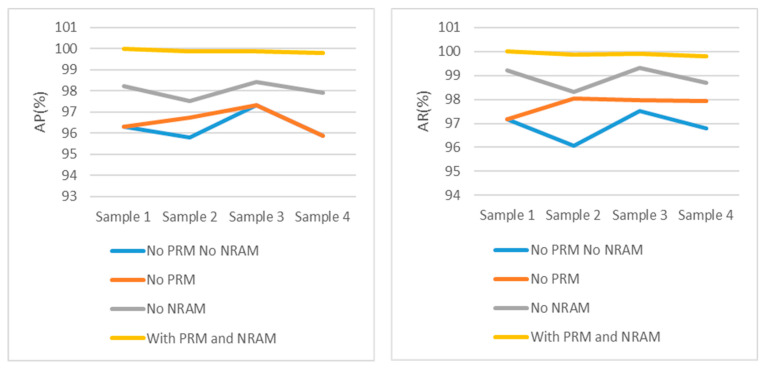
Experimental results data for four samples.

From Table 3 and Figure 10, it can be seen that DYNet using only STSM obtained the best AP value of 97.31% and AR value of 97.53% in sample 3.

Due to the high noise in samples 2 and 4, NRAM had a greater performance improvement in these two samples. However, due to the poor lighting conditions of sample 4, it was difficult for NRAM to produce satisfactory results without RPM.

When RPM was added, DYNet achieved a minimum performance improvement of 1.12% for all samples compared to the basic version. DYNet achieved a minimum of 99.87% performance for all samples when RPM and NRAM acted together.

### 5.4. Performance Comparison

**Figure 11:**
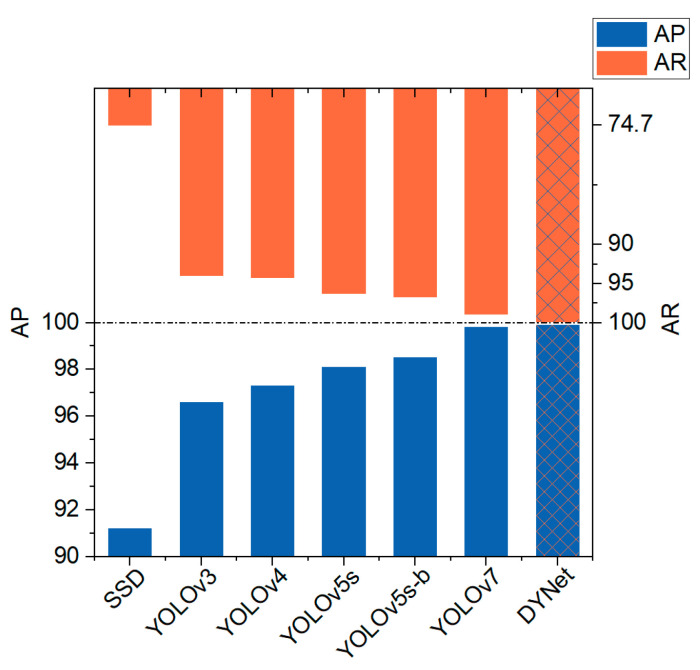
Performance comparison.

**Figure 12:**
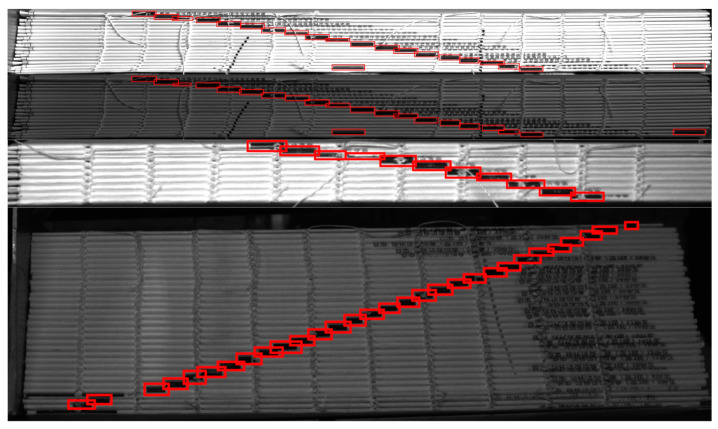
Detection results of the four small-target samples (top to bottom: samples 1–4).

DYNet is based on the Darknet framework for the detection of book ladder markers, so in this study, we chose to compare the more novel models among the homologous target detection algorithms using the Darknet framework. The comparison results between DYNet and other target detection methods of the same type for four small-target samples are shown in Table 4 and Figure 11. The detection results of DYNet for each target are shown in Figure 12. This study noted that YOLOv8 has been published, but unfortunately the Darknet framework does not support the YOLOv8 algorithm, and it is therefore not included in the comparison.

As shown in Table 4, AP improved by 8.7% and AR by 25.2% compared to SSD. Compared to YOLOv3, YOLOv4, and YOLOv5s, AP improved by 3.3%, 2.6%, and 1.8%, and AR improved by 6%, 5.7%, and 3.7%, respectively. Compared with the improved YOLOv5s (YOLOv5s-b in the table) proposed by Yang [9] et al. in 2022, AP improved by 1.4% and AR by 3.2%. Compared to the YOLOv7 proposed by Wang [14] et al. in 2022, AP improved by 0.1% and AR improved by 1%.

As can be seen from the table, with the continuous optimization and iteration of the model, its AP and AR performances improved significantly. The performance of the SSD model was relatively weak. The YOLOv3 and YOLOv4 models reached a high level of performance. The YOLOv5s and improved YOLOv5s models further improved the detection performance, with AP and AR values exceeding 98%. The YOLOv7 model achieved an impressive performance, with AP and AR values reaching 99.8% and 98.9%, respectively. Finally, the AP and AR values of the DYNet model were both 99.9%.

## 6. Conclusions

To realize real-time, fast, and accurate detection, this paper proposed the high-precision DYNet detection model for small targets based on the characteristics of the ladder label dataset. It referenced a two-stage target detection algorithm that integrated the functions of extracting object regions and classifying and recognizing regions in one model. DYNet was used to perform comparative tests on four small-target samples, and a quality evaluator was designed. The output of the quality evaluator was used as an evaluation indicator for model performance. The results show that DYNet had better detection accuracy for small targets compared to other models.

The dataset was the main factor affecting the CNN-based target detection algorithm. In this study, we used a self-built dataset as the training samples and achieved high detection accuracy during the testing process. However, DYNet will be confronted with certain challenges in dealing with special data samples that may appear in the future. Thus, it is still necessary to perform a more comprehensive evaluation and optimization of its performance and to further explore its robustness in different environments or scenarios. For target detection of different types, sizes, and angles, the adaptability of this model may be limited.

## 7. Discussion

The detection accuracy and recall rate of the DYNet high-precision detection model proposed in this paper meet actual production needs. However, because DYNet is composed of two one-stage object detection algorithms, its detection speed was slightly inferior to that of a single one-stage object detection algorithm. In practical production, DYNet can efficiently detect targets in most cases. However, there may be some delay when processing high-speed image data. This is unacceptable for real-time applications or scenarios that require a quick response.

To optimize the adaptability and detection speed of the DYNet high-precision detection model, the following measures will be considered to improve DYNet in the future:

1. Introduce more samples and data enhancement techniques: By introducing more types, sizes, and angles of target samples and combining data enhancement techniques, DYNet’s adaptability to various target detection situations can be enhanced.
2. Optimize model architecture and algorithms: By adjusting and improving the model architecture and using more advanced target detection algorithms, the accuracy of the algorithms and processing speed can be improved.

## Figures and Tables

**Table 1:** Detailed information of the four small-target samples.

Sample No.	Image Size	Quantity	Details of the Target and the Background
1	416 × 416	1133	With light, low noise
2	416 × 416	764	With light, high noise
3	416 × 416	891	No light, low noise
4	416 × 416	532	No light, high noise

**Table 2:** Sources of small-target samples.

The Title of the Book	Quantity
Dictionary of Common-Used Ancient Chinese Words	1330
Xi Jinping: The Governance of China	1234
Modern Chinese Dictionary	313
Les Misérables	103
The Brain Project	76
Notre Dame de Paris	62
Zero to One: Notes on Startups, or How to Build the Future	60
World Order	55
Complete Growth	50
<1942>	30
The Past	29
Total	3342

**Table 3:** Ablation experiment.

RPM	NRAM	Sample 1		Sample 2		Sample 3		Sample 4	
		AP (%)	AR (%)	AP (%)	AR (%)	AP (%)	AR (%)	AP (%)	AR (%)
✘	✘	96.29	97.18	95.81	96.07	97.31	97.53	95.86	96.80
✘	✔	96.29	97.18	96.73	98.04	97.31	97.98	95.86	97.93
✔	✘	98.23	99.21	97.51	98.30	98.43	99.33	97.93	98.68
✔	✔	**100.00**	**100.00**	**99.87**	**99.87**	**99.89**	**99.89**	**99.81**	**99.81**

**Table 4:** Performance comparison.

Model	Iteration	Backbone	AP	AR
SSD [11]	12 k	VGG16	91.2	74.7
YOLOv3 [12]	12 k	Darknet-53	96.6	93.9
YOLOv4 [13]	12 k	CSPDarknet-53	97.3	94.2
YOLOv5s	12 k	CSPDarknet-lite	98.1	96.2
YOLOv5s-b [9]	12 k	CSPDarknet-lite	98.5	96.7
YOLOv7 [14]	12 k	ResNet-50	99.8	98.9
**DYNet (*Ours*)**	**12 k**	CSPDarknet-53	**99.9**	**99.9**

## Data Availability

Data underlying the results used in this paper are not publicly available at this time but may be obtained from the authors upon reasonable request.
